# Spatial–Temporal Features of Coordination Relationship between Regional Urbanization and Rail Transit—A Case Study of Beijing

**DOI:** 10.3390/ijerph19010212

**Published:** 2021-12-25

**Authors:** Xuanxuan Xia, Hongchang Li, Xujuan Kuang, Jack Strauss

**Affiliations:** 1 School of Economics and Management, Beijing Jiaotong University, Beijing 100044, China; 20113017@bjtu.edu.cn; 2 Civil Aviation Management Institute of China, Beijing 100102, China; ivy.kuang@camic.cn; 3 Reiman School of Finance, University of Denver, Denver, CO 80208, USA; jack.strauss@du.edu

**Keywords:** regional urbanization, rail transit, coupling coordination degree, spatial–temporal features

## Abstract

Urban rail transit is an important transportation infrastructure that mitigates the congestion of the central city and realizes compact city space development. However, the literature on the spatiotemporal coupling of urbanization and rail transit from the urban scale and its influencing factors is still uncommon. Taking Beijing as an example, based on the theory of coupling coordination, we have constructed a comprehensive indicator system for regional urbanization (hereafter RU) (including population, economy, and spatial urbanization) and rail transit (hereafter RT). On this basis, we use the entropy method, coupling coordination degree model, and spatial autocorrelation analysis method to explore the spatiotemporal characteristics of the overall and pairwise coupling coordination between population, economy, spatial urbanization, and rail transit. Finally, we analyze the spatial correlation and standard deviational ellipse analysis of the coupling coordination degree between RU and RT. The results indicate the following: (1) In addition to population urbanization, the other urbanization indicators and the RT level all show a downward–rising–falling trend from 2006 to 2017, among which the level of economic urbanization is the highest. The degree of coupling coordination between RU and RT is unbalanced development and shows a trend of first rising and then falling. (2) The degree of coupling coordination between RU and RT presents an imbalanced distribution in various regions, and the coupling coordination degree in the central urban areas is significantly higher than that in the outer suburbs. (3) From 2006 to 2017, the spatial correlation of the coupling coordination degree between the various systems has a similar changing trend. Moreover, the distribution of the spatial agglomeration points of the coupling coordination degree between RU and the RT is similar, showing a decreasing trend from the central urban area to the surrounding urban area. Therefore, relevant departments can rationally plan the construction of urban rail transit according to the coordination relationship between RU and RT and the spatial aggregation degree to realize the benign and sustainable development between urban especially suburbanization and rail transit.

## 1. Introduction

In recent years, with the rapid development of the economy, urbanization and motorization have further accelerated, the urban population continues to increase, and the scope of urban scale is expanding. As of the end of 2020, China’s urbanization level exceeded 60%. However, with the speed of urbanization, it is a series of urban issues, such as road traffic congestion, unreasonable urban spatial structure, and waste of resources. The traffic problems of the city have always existed, but urban traffic contradictions are becoming more serious, and the unreasonable structural defects in the large urban spatial layout are more fully sufficient. For example, according to data statistics, Beijing’s annual traffic congestion is direct; indirect economic losses reaches tens of thousands of RMB, which probably accounts for 5% of Beijing’s GDP. The problems caused by air quality and friction disputes have also affected the quality of life of thousands of people, which has become a social problem that cannot be ignored. To support urban development and construction as well as the convenient travel of residents, many cities have begun to plan rail transit networks. As an important part of the urban public transportation system, urban rail transit is an important part of its shipping, speed, convenience, and punctuality, which solves one of the important tools of the city mayor. At the same time, the economic role of rail transit is also very significant, with the rail transit-oriented urban development model being an important trend in many world cities. Vigorously developing fast, environmentally friendly, economical urban rail transit has become the key to accelerating urbanization processes and resolving traffic contradictions in major cities. According to the data released by the Ministry of Transport, as of 31 December 2020, there were 44 cities in China to operate urban rail transit, which accounts for 6.29% of China’s total cities. This also depends to some extent on China’s urban rail transit systems still needing further rapid development. Among them, the planning and construction of the comprehensive transportation hub are strengthened, and the urban rail transit is axial, thereby achieving a comprehensive improvement of urban traffic congestion, improving working efficiency and the quality of urban life.

On 19 January 2021, the National Development and Reform Commission of China held a press conference, indicating that China will focus on “two new” and shortboard weaknesses. It stated that China should speed up the construction of rail transit, gradually improve the urban rail transit network, and strive to establish a modern urban comprehensive transportation system with public transportation network as the main body and rapid rail transit as the backbone, which is in line with the process of urban development. Within the city, there is also a significant uncoordinated issue between rail transit and regional urbanization. For example, the urban rail construction in the central city is perfect, while in the outer suburbs, it is relatively deficient. In 2000, only Beijing, Tianjin, Shanghai, and Guangzhou had urban rail transit lines. The urban rail lines are mainly distributed in the city center, and there are relatively few in the outer suburbs. For example, the urban rail lines in Beijing only cover 11 administrative regions, and there are at least six urban rail lines in the central city, while in the outer suburbs such as Huairou, there are fewer than two. Therefore, in the process of urban construction, the coordinated development of urbanization and urban rail in different regions is important.

## 2. Literature Review

The coordination and sustainability of urbanization have always been the focus of scholars. In the process of urbanization development, rail transit construction and urbanization development are mutually promoting, so the coordinated development between them is important. The existing research can be divided into two categories: qualitative research and quantitative research. For the former, scholars mainly take the perspective of whether the rail line optimization, rail station optimization, and comprehensive hub transfer mode are coordinated with urbanization. In addition, quantitative analysis mainly uses the quadrant graph, coupling degree model, Theil coefficient model, and spatial analysis method to analyze the coordination between rail transit and urbanization-related factors.

### 2.1. The Impact of Rail Transit on the Urbanization Development

Rail transit planning has gradually become an important part of urban construction, and rail transit construction has a significant promoting effect on urban economic growth [1]. Firstly, the value appreciation of land around rail lines is particularly significant [2,3,4,5,6,7]. For example, Kim and Zhang have studied the impact of rail transit on commercial land, with the positive impact of traffic trend of commercial land in Seoul, South Korea, but there is a difference in premium between the locations [8]. According to the report of Pacheco-Raguz, the land value near Metro Line 1 in Milan, Italy has increased slightly [9]. KO and CAO have found that Hiawatha rail transit in Minneapolis, Minnesota promoted commercial real estate prices rising to $24.6 per square foot [10]. Secondly, scholars have optimized and analyzed the existing rail lines and stations [11], determined the stations and periods with high traffic pressure, put forward optimization plans, and considered the passenger flow capacity of a single station or an interval in terms of network structure [12]. From the three aspects of structural dimension, utilization dimension, and operation dimension, the development strategy of the three-in-one public space of a subway station is put forward [13]. Researchers also analyzed the change of the number of rail transit passengers and the transfer time spent in the hub region [14], thereby proposing the transfer mode of an urban rail transit system integrated hub, which can effectively shorten the travel time and relieve urban traffic pressure [15], which promotes the development of urbanization to a certain extent. In general, regarding the largest urban transportation and land impact, the most stressed station is called the urban rail transit hub [16], which is because there are important functional areas such as business districts and residential areas. Therefore, the construction of facilities is mainly concentrated in the main road rather than the branch, causing the structural proportion of the peripheral roads around the traffic hub [17,18], which affects the level of urbanization. Although rail transit and urban coordinated development can bring benefits to the urban economy, transportation, society, and the environment [19], due to inconsistency in the actual development process, the efficiency of rail transit is low [20,21]. Therefore, many scholars emphasize the study of coordinated development levels [19,22].

Previous studies mainly focused on the influence of rail transit construction planning on urbanization development, but they did not analyze and study from the direction of coordination between the two. Urbanization development is a complex process that we need to analyze from different angles. This paper studies the coordination between rail transit and urbanization and the related influencing factors from three perspectives of economic urbanization, population urbanization, and spatial urbanization, so as to provide some theoretical support for the healthy development of urban track and urbanization.

### 2.2. Based on Different Quantitative Analysis Methods, the Relevant Factors Affecting the Urbanization Development Level Are Studied

Firstly, scholars have carried out multi-dimensional studies on the relationship between rail transit and urbanization development. For example, Hou has analyzed the coordination relationship between rail transit and land use based on multivariate data through data envelopment analysis (DEA) and the clustering method, finding that the relationship between rail transit capacity and land use of rail transit stations with high population density is unbalanced [23]. Liu has calculated the comprehensive development level of rail transit system and land use according to the utility function and built a coupling coordination degree model to quantitatively analyze the coordination of Shanghai rail transit and land use [24]. Cai has proposed an urban rail transit model by analyzing the urban spatial structure and land distribution characteristics, putting forward corresponding technical suggestions based on foreign urban rail transit network systems and corresponding connection modes [25]. From the perspective of spatial overlap between rail transit stations and urban centers, the principle of spatial coupling is expounded, and it is pointed out that rail transit will have a significant or fundamental impact on the urban spatial structure, land use, and spatial quality [26,27]. In addition, emerging techniques such as data mining [28,29], computer application processing [29,30], and intelligent transport [31,32,33,34] have also been applied in analyzing the relationship between rail transit and urbanization.

Secondly, scholars also have studied the factors influencing urbanization from the aspects of urban functional mixing degree, population, land, and so on. Based on information entropy and coupling coordination degree, the coordination relationship between urban function mixing state and urbanization development level and its influencing factors are analyzed from the perspective of urban spatial structure development [35]. Cao has studied the coordination of population, land, and economic urbanization from the perspective of system theory [36]. Zhang has conducted a quantitative study on the coordination between urbanization quality and scale in Jiangsu Province by using the quadrant method [37]. Li has used the coupling model to study the coordination of urbanization in Chongqing from the three dimensions of land, population, and industry [38]. Li has used the Theil coefficient to measure the urbanization coordination of 105 prefecture-level cities in the Yangtze River Economic Belt from the aspects of urban and rural areas, industrial cities, regional urbanization, and environmental resources [39]. However, urbanization is a complex system, coordination is a comprehensive concept, and the research direction of scholars is not completely consistent. For example, Tian has used a coupled coordination degree model and spatial autocorrelation statistical model to evaluate the spatiotemporal coupling and interaction between ecosystem and urbanization, and to understand the coupling relationship between urbanization and ecological protection [40]. Li has analyzed the relationship between urbanization level and ecological environment in Lianyungang city from 2000 to 2008 and found that the coupling coordination degree between urbanization level and ecological environment presents a U-shaped curve over time [41]. Wang has found that the coupling coordination degree between population urbanization and ecological environment in Beijing–Tianjin–Hebei presents an S-shaped curve over time [42]. Zhao has proposed a new compactness measurement method and explored the relationship between compactness and urbanization using GWR model and spatial autocorrelation analysis [43]. Ma has analyzed the development coordination degree of cities in the middle reaches of the Yangtze River from three aspects of economy, society, and ecology, and analyzed the differences among different regions [44].

Most of the existing studies focus on one aspect of the system elements in the process of urbanization from a certain perspective, and all of them are from the perspective of the city as a whole. Few studies have quantified and measured the coordinated development relationship between urban orbit and urbanization from the perspectives of economic urbanization, population urbanization, and spatial urbanization by taking different regions of a city as research units. The specific synchronization research comparison can be shown in Table 1. Due to the economy, infrastructure, population, and various policies of different parts of the city, this leads to differences in the urbanization process. Therefore, urban rail planning strategies and urbanization development policies in different urban areas need to adapt to local conditions. Therefore, based on the coupling coordination degree model and spatial autocorrelation model, we study and analyze the coordinated development relationship between rail transit and urbanization for different areas within the city. This enriches the theoretical basis between rail transit and urbanization development to some extent and makes quantitative and spatial analysis on the coordination between them.

To sum up, previous studies on the relationship between rail transit and urbanization mainly focus on the degree of mutual influence and promoting effect, and the coupling relationship between rail transit and urban population, space, and industrial structure. However, most of the previous studies have studied the causes of the imbalance between rail transit and urbanization development in different cities from the perspective of the city as a whole, and studies on the factors of the imbalance between rail transit and urbanization development from the perspective of urban internal regions remain rare. Compared with similar previous studies, our innovation is mainly reflected in the following two aspects. First, based on the coupling coordination model, we measure the coordination relationship among the economic, spatial, and population urbanization and rail transit as a whole, as well as pairwise. Second, we explore the internal reasons that affect the unbalanced development of urban regions by looking at the coordination relationship between rail transit and the urbanization level. Based on the data of rail transit and urbanization development level from 2006 to 2017, we construct a comprehensive indicator system of regional urbanization (population, economic, spatial urbanization) and rail transit, and we calculate the coupling coordination degree between the urbanization and rail transit of each region in Beijing. Finally, based on the spatial autocorrelation model, the associated characteristics of the coupling coordination in space are analyzed. According to the research results, relevant departments can make corresponding decisions on urban rail line planning, reasonably plan the urban space layout, and provide theoretical support for urban optimization management and sustainable development.

## 3. Methodology

### 3.1. Study Area

**Figure 1:**
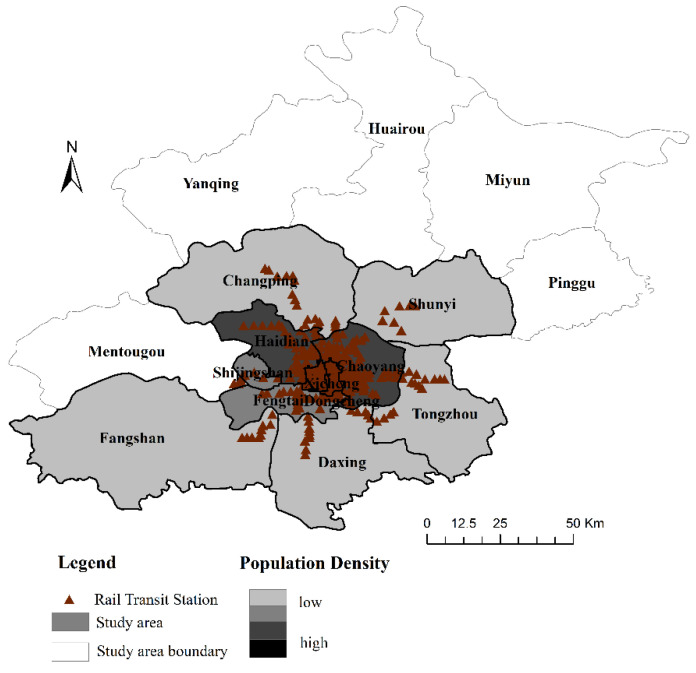
Distribution of Beijing Rail Transit Lines.

Beijing has a certain representation as China’s political, economic, technology, and transportation center. Taking Beijing as an example, we analyze the coordination relationship between regional urbanization and rail transit. With the rapid development of rail transit in China, the subway is not only the main form of urban rail transit but also the focus of urban rail transit construction in the future. As of January 2017, Beijing has a total of 19 subway lines covering 11 municipal districts, mainly including Dongcheng, Xicheng, Haidian, Chaoyang, Fengtai, Shijingshan, Changping, Fangshan, Tongzhou, Shunyi, and Daxing. Based on the actual operation of the Beijing subway, the research area of this paper is shown in Figure 1. The research data mainly comes from the “Beijing Statistical Yearbook” and “Beijing Regional Statistical Yearbook” in 2007–2018.

### 3.2. Construction of the Evaluation Indicator System

Referring to the indicators of urbanization and rail transit at home and abroad, the indicator system of this paper mainly includes two aspects: regional urbanization and rail transit. According to the relevant regulations and description of urbanization by the Institute of Urban Environment, Chinese Academy of Sciences, urbanization is a multidimensional concept, including population urbanization, economic urbanization, and spatial urbanization. Since urbanization is a complex process, demography defines urbanization as the process of rural population transforming into urban population, economics defines urbanization from the perspective of economic model and mode of production, and geography defines urbanization as the process of rural areas or natural areas transforming into urban areas. Therefore, we select the corresponding indicator data from three aspects of population urbanization, economic urbanization, and spatial urbanization to measure the RU. Firstly, we construct the indicator system of regional urbanization from the three criteria layers of population urbanization, economic urbanization, and spatial urbanization [40]. Secondly, due to the limitation of data, the rail transit system selects the number of stations and subway lines serving the region and other indicators to measure the development level of rail transit in different regions. Based on the principles of systematization, scientificity, comprehensiveness, and operability of the indicator system construction, we selected 22 indicator layers altogether. The specific indicator system and weight are shown in Table 2 [40,45,46].

### 3.3. Methods

#### 3.3.1. The Coupling Coordination Degree Model

The coupling degree refers to the phenomenon of two (or more) systems that affect each other by various interactions, including the promotion system from disordered ordered processes, which describes the interaction between systems or elements. The coupling coordination refers to the high and low level of the interaction between the two systems or the multi-system, as well as the uniformity of the benign association between the various elements within the system, indicating the tendency between the system from disordered to order. To study the coordination relationship between regional urbanization and urban orbit, we selected four systems with population urbanization, economic urbanization, spatial urbanization, and rail transit. Then, based on the coupling coordination model, we measure the coupling degree and coupling coordination between the four systems, and the specific calculation steps are as follows:

Step 1: Measure the levels of each system. We integrate various types of evaluation methods, considering the lack of objective basis in the determination of the weight in the subjective empowerment evaluation method. In some cases, the subjective judgment may have a certain deviation. As an objective evaluation method, the entropy method can not only avoid the subjective judgment of the researcher but also solve the information overlap problems between multiple indicators [45,46]. Therefore, according to the various evaluation indicator systems given in Table 1, we use entropy values to determine the weight of each indicator (the results shown in Table 1), and the steps are as follows:

(1) Standardized processing. This type of processing is used to eliminate the inconsistency of the measurement units of each indicator, the size of the measurement units, and the direction of the positive and negative direction. Therefore, the data are standardized before calculating the comprehensive indicator, we homogenized the indicators of different qualities, and their value ranges are [0, 1]. Standardization of the forward indicators and negative indicators is shown in Equations (1) and (2), respectively.

Positive indicator:

(1) $$X_{ij}^{\prime }=\frac{X_{ij}-\min\left\{X_{1j},\cdots ,X_{11j}\right\}}{\max\left\{X_{1j},\cdots ,X_{11j}\right\}-\min\left\{X_{1j},\cdots ,X_{11j}\right\}}$$

Negative indicator:

(2) $$X_{ij}^{\prime }=\frac{X_{ij}-\max\left\{X_{1j},\cdots ,X_{11j}\right\}}{\max\left\{X_{1j},\cdots ,X_{11j}\right\}-\min\left\{X_{1j},\cdots ,X_{11j}\right\}}$$

where $X_{ij}$ and $X_{ij}^{\prime }$ respectively represent the value of the indicator j and the standardized indicator value in the region i. $\max\left\{X_{1j},\cdots ,X_{11,j}\right\}$ and $\min\left\{X_{1j},\cdots ,X_{11,j}\right\}$ represent the maximum and minimum values of the indicator j in all regions (i=1,2,⋯,11; j=1,2,⋯,22).

(2) Measure the level of each system. Firstly, we calculate the information entropy $e_{j}$ of indicator j, as shown in Equation (3):

(3) $$e_{j}=-\frac{1}{\ln11}\sum_{i=1}^{11}P_{ij}\times \ln P_{ij}\left(0\leq e\leq 1\right).$$

Here, $P_{ij}$ indicates the weight of indicator j in region i. The calculation formula is $P_{ij}=X_{ij}^{\prime }/\sum_{i=1}^{11}X_{ij}^{\prime }$. Then, the level of each system in region i is calculated, as shown in Equation (4):

(4) $$U_{i}=\sum_{j=1}^{22}w_{j}\times X_{ij}^{\prime }.$$

Here, $w_{j}$ is the weight of indicator j, and the calculation formula is $w_{j}=\left(1-e_{j}\right)/\sum_{j=1}^{22}\left(1-e_{j}\right)$.

Step 2: Calculate the coupling degree between urbanization and urban orbit in region i, as shown in Equation (5):

(5) Ci={Up×UE×US×UR[(UP+UE+US+UR)/4]4}14

where $U_{p},\;U_{E},\;U_{S}$ and $U_{R}$ represent the level of population urbanization, economic urbanization, spatial urbanization, and rail transit, respectively.

To further explore the coupling relationship between population urbanization, economic urbanization, spatial urbanization, and rail transit in region i, the calculation formula is shown in Equation (6):

(6) $$C_{i}^{PR}=\frac{2\sqrt{U_{P}\times U_{R}}}{U_{P}+U_{R}},\;C_{i}^{ER}=\frac{2\sqrt{U_{E}\times U_{R}}}{U_{E}+U_{R}},\;C_{i}^{SR}=\frac{2\sqrt{U_{S}\times U_{R}}}{U_{S}+U_{R}}.$$

To judge the coupling degree of the three systems or two systems, the coupling degree is divided into four levels based on previous research results, as shown in Table 3 [47].

Step 3: Calculate the coupling coordination degree between RU and RT in the region i, as shown in Equation (7):

(7) $$D_{i}=\sqrt{C_{i}\times T_{i}},T_{i}=\alpha U_{P}+\beta U_{E}+\varphi U_{S}+\gamma U_{R}$$

where $T_{i}$ is the comprehensive evaluation indicator of the four systems in region i. α, β, φ, γ is the undetermined coefficient and satisfies α+β+φ+γ=1. RT and RU promote each other, and their development plays a complementary role. In this paper, RU is divided into economic urbanization, population urbanization, and spatial urbanization. Referring to the relevant literature [24,45], each system is equally important when calculating the interaction between the two, so the coefficients in Equation (7) are set as 0.25, that is α=β=φ=γ=0.25.

Similarly, there are pairwise coupling coordination relationships among population urbanization, economic urbanization, spatial urbanization, and rail transit. The calculation formula is shown in Equation (8):

(8) $$D_{i}^{PR}=\sqrt{C_{i}^{PR}\times T_{i}^{PR}},\;D_{i}^{ER}=\sqrt{C_{i}^{ER}\times T_{i}^{ER}},\;D_{i}^{SR}=\sqrt{C_{i}^{SR}\times T_{i}^{SR}}.$$

Here, $D_{i}^{PR}$ is the degree of coupling coordination between population urbanization and rail transit, at this time, $T_{i}^{PR}=\alpha U_{i}^{P}+\gamma U_{i}^{R}$. $D_{i}^{ER}$ is the degree of coupling coordination between economic urbanization and rail transit, at this time, $T_{i}^{ER}=\beta U_{i}^{E}+\gamma U_{i}^{R}$. $D_{i}^{SR}$ is the degree of coupling coordination between spatial urbanization and rail transit, at this time, $T_{i}^{SR}=\varphi U_{i}^{S}+\gamma U_{i}^{R}$. Referring to the relevant literature [24,45], the RT and RU promote and influence each other. When studying the coordination relationship between population urbanization, economic urbanization, spatial urbanization, and urban rail respectively, the two systems are considered as equally important for calculation. Thus, the influence of certain subjective factors can be excluded to some extent, so the coefficient is set as 0.5, that is α=β=φ=γ=0.5.

The classification is based on the degree of coupling coordination, and the specific classification criteria are shown in Table 4 [48].

#### 3.3.2. Spatial Autocorrelation Analysis

##### Global Spatial Autocorrelation Model

The Moran’s I indicator is used to calculate the degree of global autocorrelation [49], and further to analyze the spatial correlation and agglomeration of the RU and the RT. The calculation formula of the Moran’s I indicator is as follows:

(9) $$I=\frac{n\sum_{i=1}^{n}\sum_{i{}^{\prime }\neq i}^{n}w_{ii{}^{\prime }}(D_{i}-\bar{D})(D_{i{}^{\prime }}-\bar{D})}{\sum_{i=1}^{n}\sum_{i{}^{\prime }\neq i}^{n}w_{ii{}^{\prime }}\sum_{i=1}^{n}{\left(D_{i}-\bar{D}\right)}^{2}}$$

where n is the number of regional units in the area, that is, the total number of administrative districts with rail transit (n=11). $D_{i},\;D_{i{}^{\prime }}$ are the coupling coordination degree of the region i and region $i^{\prime }$ $\left(i=1,2,\ldots ,11,\;i\neq i^{\prime }\right)$, respectively. $\bar{D}$ is the mean value of the coupling coordination degree. $w_{ii{}^{\prime }}$ is the distance weighted of the region i and region $i^{\prime }$. If 0<I≤1, it indicates that the degree of coupling coordination of each region is positively correlated; if −1≤I<0, there is a negative correlation; if I=0, there is no spatial correlation.

For Moran’s I indicator, standardized statistics Z are generally used to test the significance of spatial autocorrelation. Its calculation formula is as follows:

(10) $$Z=\frac{I-E\left(I\right)}{\sqrt{VAR\left(I\right)}}$$

where E(I) and VAR(I) are the theoretical expectation and theoretical variance of I, respectively.

If Z is positive and significant, it will indicate that there is a significant positive correlation; that is, similar observations tend to gather in space. If Z is negative and significant, it will indicate that there is a significant negative correlation; that is, similar observed values tend to be dispersed. If the Z value is 0, the observed values are distributed independently and randomly.

##### Local Spatial Autocorrelation Model

By calculating the $Geti-Ord\;Gi^{*}$ indicator, the high and low value distribution of the coupling coordination degree can be identified in the process of evolution. The calculation formula is as follows:

(11) $$G_{i}^{*}=\frac{\sum_{i^{\prime }=1}^{n}w_{ii^{\prime }}D_{i^{\prime }}}{\sum_{i=1}^{n}D_{i}}.$$

At the same time, there are corresponding standardized statistics $Z{\left(i\right)}^{*}$ for the indicator $G_{i}^{*}$:

(12) $$Z{\left(i\right)}^{*}=\frac{G_{i}^{*}-E\left[G_{i}^{*}\right]}{\sqrt{VAR\left[G_{i}^{*}\right]}}$$

where $E\left[G_{i}^{*}\right]$ and $\sqrt{VAR\left[G_{i}^{*}\right]}$ are the expectation and variance of $G_{i}^{*}$, respectively. If the value of $Z{\left(i\right)}^{*}$ is positive and significant, it will indicate that the region around i is a cluster of high-value space. If the value of $Z{\left(i\right)}^{*}$ is negative and significant, it will indicate that the region around i is a low-value spatial agglomeration.

#### 3.3.3. The Models for Pattern Evolution Analysis

The standard deviational ellipse method proposed by Professor Lefever in 1926 is one of the classical methods to analyze the directional characteristics of spatial distribution. This model takes the mean of x and y coordinates of all elements to calculate the mean center, which is taken as the starting point. Then, it calculates the standard deviation of x and y coordinates to define the axis of the ellipse containing the distribution of all elements [50]. The calculation of the standard deviational ellipse includes rotation angle *θ*, the standard deviation of the long axis, the standard deviation of the short axis, and the center point. Among them, the long axis represents the direction of the data distribution, the short axis represents the range and dispersion of the data distribution, and the center point represents the center of gravity of the space element layout. The standard deviational ellipse method can identify the direction and distribution trend of data. We analyze the spatial evolution of the coupling coordination between RU and RT, which can understand the evolution of coordination between urbanization and rail transit. The calculation steps of the standard deviational ellipse method are as follows.

Step 1. Determine the ellipse center ($SDE_{x}$, $SDE_{y}$), as shown in Equation (13):

(13) $$SDE_{x}=\sqrt{\frac{\sum_{i=1}^{m}{\left(x_{i}-\bar{x}\right)}^{2}}{m}},\;SDE_{y}=\sqrt{\frac{\sum_{i=1}^{m}{\left(y_{i}-\bar{y}\right)}^{2}}{m}}.$$

Here, $x_{i}$, $y_{i}$ represent the spatial coordinate position of each element. $\bar{x}$ and $\bar{y}$ represent the arithmetic mean center, and m is the total number of all point elements.

Step 2. Calculate the rotation angle θ. Based on the x axis, the north direction is 0 degrees and rotates clockwise. The specific Equation (14) is as follows:

(14) $$\tan\theta =\frac{\left(\sum_{i=1}^{m}{\bar{\bar{x}}}_{i}^{2}-\sum_{i=1}^{m}{\bar{\bar{y}}}_{i}^{2}\right)+\sqrt{{\left(\sum_{i=1}^{m}{\bar{\bar{x}}}_{i}^{2}-\sum_{i=1}^{m}{\bar{\bar{y}}}_{i}^{2}\right)}^{2}+4{\left(\sum_{i=1}^{m}{\bar{\bar{x}}}_{i}{\bar{\bar{y}}}_{i}\right)}^{2}}}{2\sum_{i=1}^{m}{\bar{\bar{x}}}_{i}{\bar{\bar{y}}}_{i}}$$

where ${\bar{\bar{x}}}_{i}$, ${\bar{\bar{y}}}_{i}$ are the deviation between the coordinates of the i point element and the average center coordinates.

Step 3. Determine the length of the elliptic length axis *x* and *y*, as shown in Equation (15):

(15) $$\alpha_{x}=\sqrt{\frac{2\sum_{i=1}^{m}{\left({\bar{\bar{x}}}_{i}\cos\theta -{\bar{\bar{y}}}_{i}\sin\theta \right)}^{2}}{m}},\alpha_{y}=\sqrt{\frac{2\sum_{i=1}^{m}{\left({\bar{\bar{x}}}_{i}\sin\theta +{\bar{\bar{y}}}_{i}\cos\theta \right)}^{2}}{m}}.$$

We use the standard deviational ellipse tool provided by ArcGIS (Environmental Systems Research Institute, Inc.; Redlands, CA, USA) to conduct standard deviational ellipse analysis on the coupling coordination degree between RU and RT and the spatial aggregation degree of the coupling coordination degree. The output size was selected as “one standard deviation ellipse surface” (covering the point element set containing 68%) [51]. The standard deviation ellipse is a classical algorithm that analyzes the direction and distribution of points. It is used to measure the direction and distribution of a group of data, and an ellipse will be output in space. In this paper, the standard deviation ellipse can be used to reveal the overall characteristics of the spatial distribution of the coupling coordination degree and local autocorrelation. The spatial anisotropy of its dispersion degree is directly expressed, mainly including determining the center point, determining the rotation angle and determining the length of the X and Y axis. The center point represents the central location of the entire data. The long half-axis of the ellipse represents the direction of data distribution, while the short half-axis represents the range of data distribution. The larger the difference between the values of the long and short half-axes (the larger the flatness), the more obvious the direction of data. On the contrary, the closer the long and short half-axes are, the less directionality they are. The shorter the short half-axis is, the more obvious the centripetal force is. Conversely, the longer the short half-axis is, the more discrete the data are.

## 4. Results

### 4.1. Overview

**Figure 2:**
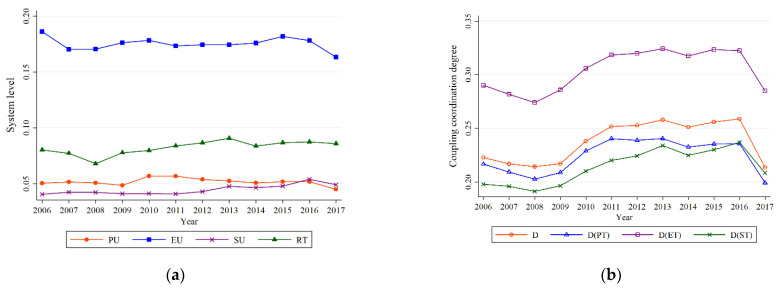
The levels of population urbanization, economy urbanization, spatial urbanization, and rail transit (**a**), the mean values of coupling coordination degree (**b**).

Urban rail transit is the key to promoting urban economic development, optimizing the urban spatial structure, and realizing sustainable urban development. At the same time, with the continuous acceleration of urbanization, the construction of urban rail transit is also constantly improving. According to Equation (4), we have obtained the levels of population urbanization (PU), economic urbanization (EU), spatial urbanization (SU), and rail transit (RT), as shown in Figure 2a. 

On the whole, from 2006 to 2017, except for PU, the changing trend of other urbanization indicators and the level of RT is consistent, which shows a downward trend from 2006 to 2008, a relatively stable rise from 2008 to 2015, and a downward trend from 2015 to 2017. In contrast, the level of PU has the highest value in 2010 but presents continuous decreasing trends from 2010 to 2017. The reasons mainly include the following three points: First, China was affected by the global financial crisis in 2008, and the economic development of Beijing showed a downward trend from 2006 to 2008. However, the 2008 Beijing Olympic Games promoted the city’s urban construction and economic development, resulting in a rising trend of urbanization indicators in Beijing from 2008 to 2015. Second, before 2008, Beijing’s urban rail construction was not perfect. In 2008, the mileage of Beijing’s rail transit reached 200 km, and 26 fast links between the new urban area and the city center, and between the city center and the suburbs were built. Therefore, urban rail transit has been on the rise since 2008. However, there has been no further development of the urban track since 2015. Third, with the removal of non-capital functions from Beijing and the construction of an “advanced and sophisticated” economic structure, employment in some traditional labor-intensive industries has decreased. According to the Blue Book of Beijing Population, Beijing’s permanent population is on the decline. For example, the number of permanent migrant residents in 2016 and 2017 dropped by 1.84 percent and 1.63 percent, respectively, compared with the previous year.

It can also be seen from Figure 2a that EU is at the highest level, followed by RT, while SU is at the lowest level. The reasons are as follows: First, Beijing is a world city and the center of China’s political, economic, cultural, and foreign exchanges. Its economy is active and developing rapidly. For example, economic urbanization indicators such as the average wage of urban employees, GDP, and urban per capita disposable income have been at a relatively high level. Second, Beijing’s urban rail transit construction is relatively perfect. According to the data, the annual passenger volume of the Beijing subway reached 4.53 billion in 2017, with an average daily passenger flow of 12.411 million, and the maximum daily passenger volume reached 13.2746 million. Third, Beijing’s space expansion is serious, but the space infrastructure is backward, such as the completed area of housing and commercial housing sales area are at a low level.

According to Equations (7) and (8), we can obtain the distribution of coupling coordination degree between RU and RT, as shown in Figure 2b. On the whole, the coupling coordination between systems is in a disordered state, and the coupling coordination between systems presents a consistent trend of change, which is shown as follows: from 2006 to 2016, the coupling coordination between systems presents an increasing trend, but from 2016 to 2017, it presents a declining trend. Secondly, the coupling coordination degree between EU and RT is at the highest level, while the coupling coordination degree between SU and RT is at the lowest level.

### 4.2. Coupling Degree Analysis

According to Equations (5) and (6), the coupling relationship between each system is obtained, and the results are shown in Table 5. The relationship between PU and RT is referred to as PT, the relationship between EU and RT is referred to as ET, the relationship between SU and RT is referred to as ST.

As can be seen from Table 4, the overall coupling degree between RU and RT is at a high level from 2006 to 2017. However, the coupling degree of PT and ET reached a high level from 2010 to 2016, and that of ST reached a high level from 2011 to 2017. This indicates that the coupling degree between urbanization and rail transit is at a high level, which indicates that each system is in a basic and highly stable state.

### 4.3. The Spatiotemporal Differentiation of Coupling Coordination

#### 4.3.1. The Spatiotemporal Differentiation of Overall Coupling Coordination Degree

**Figure 3:**
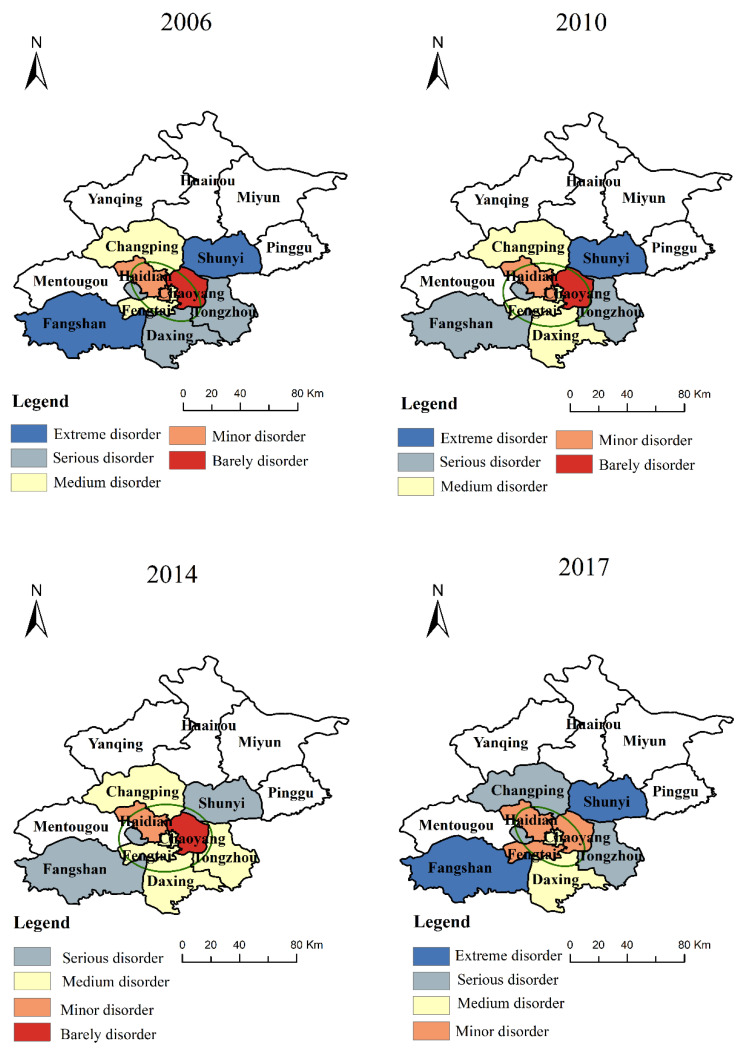
The spatiotemporal distribution of the overall coupling coordination degree.

From Figure 3, it can be found that the coupling coordination degree between RU and RT presents an imbalanced distribution in various regions, and the coupling coordination degree of the central urban area is significantly higher than that of the outer urban area. On the whole, from 2006 to 2014, the overall coupling coordination degree of the central urban area (the sixth district) does not significantly change, but the coupling coordination degree of the outer suburbs (Changping, Shunyi, Tongzhou, Daxing, Fangshan) shows an obvious upward trend. In addition, the degree of coupling coordination in each region significantly decreased in 2017. First, with the continuous strengthening of the functions of the central city and the simultaneous construction of the central city and the outer suburbs, tidal centripetal traffic intensifies. After the 2008 Beijing Olympic Games, the construction of a suburban rail transit line has been actively prepared, which has strengthened the coordination relationship between RU and RT in the outer suburbs. Second, from 2016 to 2017, both RU and RT show a downward trend, which is mainly because the growth rate of the urban core area began to lag behind that of the surrounding areas, and the growth rate of the population in Beijing significantly slowed down. In the future, population growth will mainly be in the surrounding areas of the metropolitan core area.

According to the spatial evolution pattern of the standard deviational ellipse of the coupling coordination degree between RU and RT in Figure 3, the standard deviational ellipse of the overall coupling coordination degree of each system from 2006 to 2017 is consistent, which is mainly distributed in the central urban area, Changping, and Shunyi. This shows that the central district, Changping, and Shunyi are the main bodies of coupling coordination between RU and RT. Among them, the length of the major axis and the minor axis of the ellipse is relatively small.

#### 4.3.2. Spatiotemporal Differentiation of Pairwise Coupling Coordination Degree

**Figure 4:**
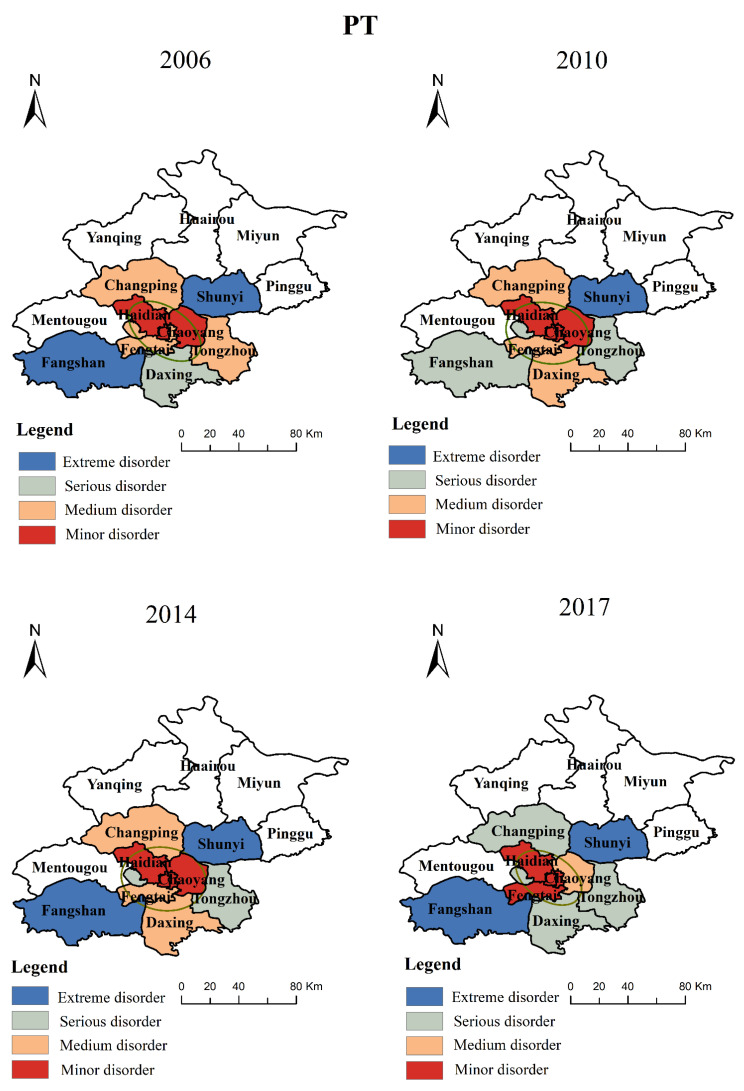

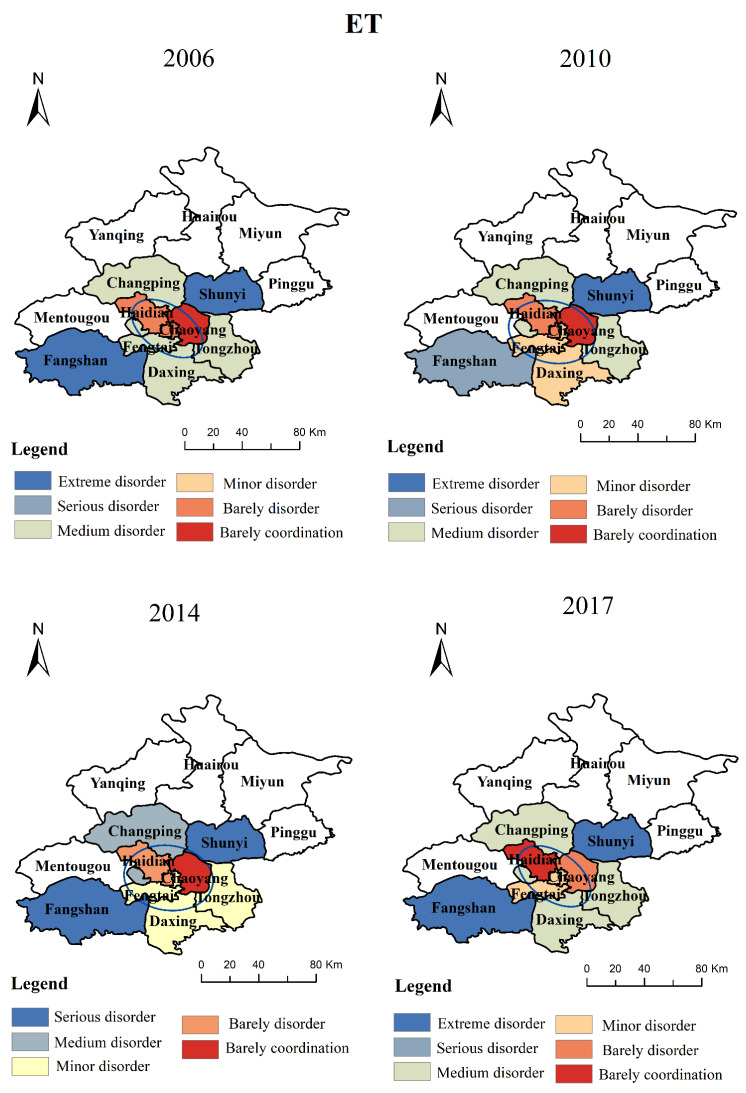

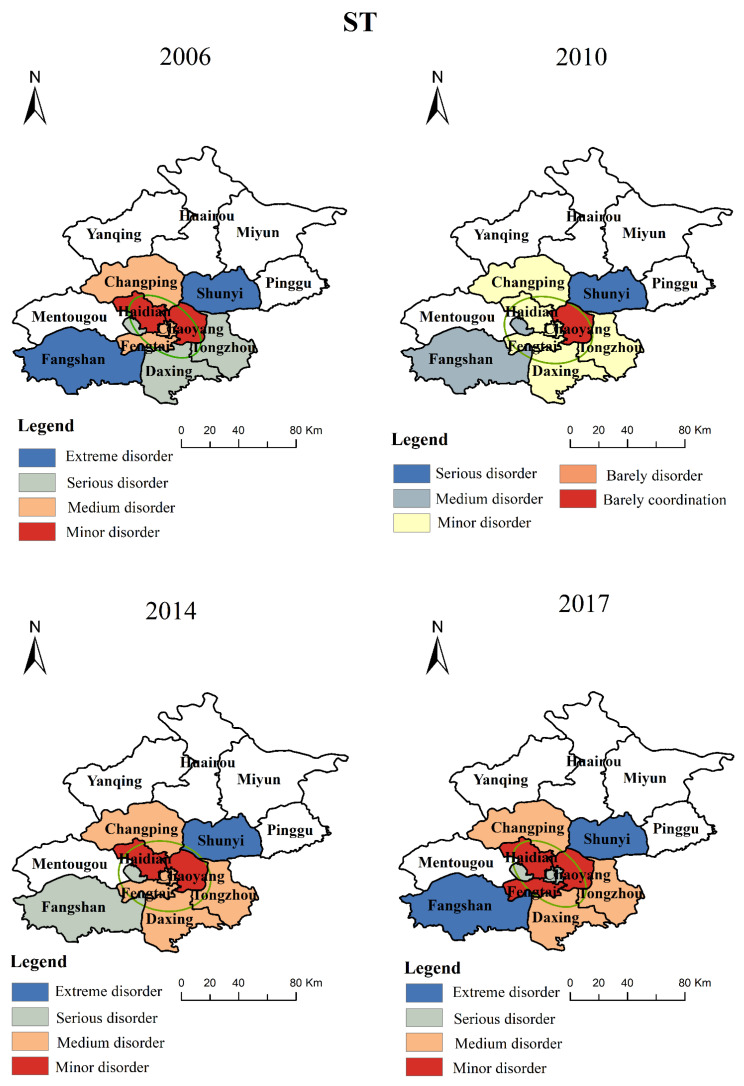
The spatiotemporal distributions of pairwise coupling coordination degree in different regions.

According to Equation (8), we obtain the pairwise coupling coordination degree of PT, ET, and ST and make spatial visualization with ArcGIS. The results are shown in Figure 4.

On the whole, the pairwise coupling coordination relationships have a significant regional imbalance, which is mainly reflected by the continuous decrease from the central city to the outer suburbs. In addition, from 2006 to 2017, the coupling coordination degree of each region shows a trend of increasing first and then decreasing. The standard ellipse of the coupling coordination degree has no obvious change, which is mainly concentrated in the central urban area, Changping, and Shunyi. This indicates to some extent that the central urban area, Changping, and Shunyi are the main areas of coordinated development between urbanization and rail transit, and there are obvious differences in the coordination relations among different regions.

From the perspective of each region, except ET, the pairwise coupling coordination relationship of PT and ST in each region is basically in a state of disorder, and the coordination relationship in the outer suburbs is almost in a state of extreme disorder or serious disorder. Firstly, the urban rail in the outer suburbs is built late, and the improvement is not as good as that in the central urban. According to data, the coverage rate of 750 m rail transit stations in the central urban reaches more than 90%, while that in Huairou and the other outer suburbs is not. Secondly, the permanent population of the central urban area has always been much higher than that of the outer suburbs. Thirdly, the construction of spatial facilities in Beijing has always been in a state of regional imbalance. For example, the spatial urbanization level of the central urban area is about 1.44 times that of the outer urban area. As can be seen from Figure 4, the coupling coordination of ET can reach barely coordination in the central urban, while it is still in a disordered state in the outer suburbs. The reason is that a large number of CBD areas are distributed in the central urban area, and the economic development has been at a relatively high level, while the economic development and rail transit of the outer suburbs have been in a state to be improved. For example, the economic level of the central urban is about 2.3 times that of the outer suburbs.

### 4.4. The Spatial Agglomeration of Coupling Coordination

#### 4.4.1. Global Spatial Autocorrelation Analysis

The distribution of spatial elements is mainly divided into three modes: agglomeration, dispersion, and enrichment. To further study the spatial distribution pattern and evolution law of D, $D^{PT}$, $D^{ET}$, and $D^{ST}$, we obtain the global autocorrelation indicators (Moran’s I and Z(I)) according to Equations (9) and (10), as shown in Table 6.

On the whole, from 2006 to 2017, the spatial correlation of the coupling coordination degree among all systems shows a similar trend, which decreased first and then increased. In addition, we can find that except for ST, the Z(I) values between other systems are positive and significant, indicating that the coupling coordination degree between systems shows a positive correlation and aggregation in space.

From Table 5, we can find that the spatial correlation indicators of the coupling coordination degree of ET and PT are all positive, which indicates that the coupling coordination degree of ET and PT is positively correlated in space. Among them, the Moran’s I value of the coupling coordination degree of PT is at the highest level; both are above 0.16. In addition, the Moran’s I value of the overall coupling coordination is negative from 2012 to 2014, indicating that there is a negative correlation between RU and RT. However, the Moran’s I values between 2006 to 2011 and 2015 to 2017 are positive and small, indicating that there is a positive spatial correlation but no obvious correlation. In addition, the global autocorrelation indicator of ST decreases from 0.088 to −0.133 from 2006 to 2016, but Moran’s I value shows an upward trend from 2016 to 2017. Although Moran’s I value is negative, it significantly increases. To a certain extent, the coupling coordination degree of ST has a strong negative spatial correlation, and the spatial difference is large.

#### 4.4.2. Local Spatial Autocorrelation Analysis

**Figure 5:**
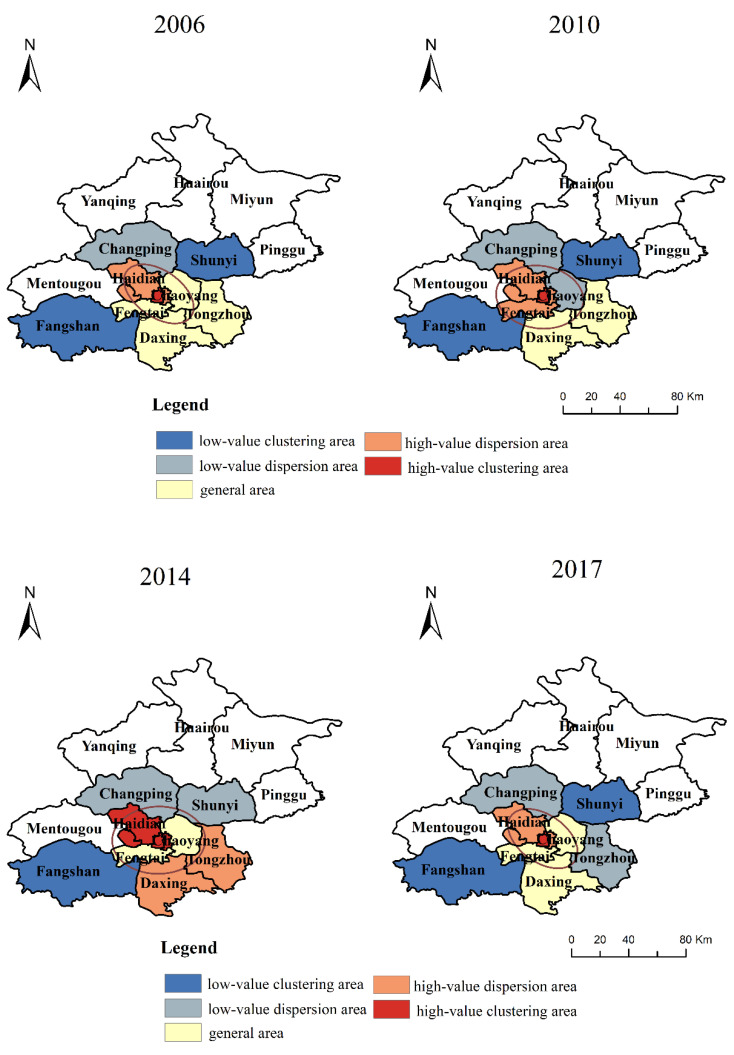
The spatiotemporal distribution of high–low value clustering points with different overall coupling coordination degrees.

To further understand the spatial aggregation between the pairwise coupling coordination degrees, we use ArcGIS to carry out regional visualization, as shown in Figure 5. On the whole, from 2006 to 2017, the spatial distribution of high and low cluster points of the overall coupling coordination degree is similar, showing a decreasing trend from the central urban to the surrounding urban. From 2006 to 2014, the number of high-value dispersion areas and high-value clustering areas of the overall coupling coordination degree increases significantly, from 4 to 6, and the aggregation state in the outer suburbs significantly increases. However, in 2017, the clustering status of each region significantly decreases. To a certain extent, it shows that there is an obvious regional imbalance in the aggregation state of the coupling coordination degree between urbanization and rail transit.

From the perspective of each region, Dongcheng, Xicheng, Haidian, and Shijingshan are high-value dispersion areas and high-value clustering areas, which belong to the region with a high coupling coordination degree and agglomeration of urbanization and rail transit. However, the coupling coordination degree of Chaoyang, Fengtai, Daxing, and Tongzhou are within the general area; Changping, Shunyi, and Fangshan are the low-value dispersion areas and low-value clustering areas of coupling coordination degree. This regional imbalance between urbanization and rail transit is mainly because the center urban development speed is higher than that in the outer suburbs. For example, the number of subway lines and subway stations in the central urban area is about 3.23 times that in the outer urban area, and the GDP of the central urban area is about 3.32 times that in the outer urban.

**Figure 6:**
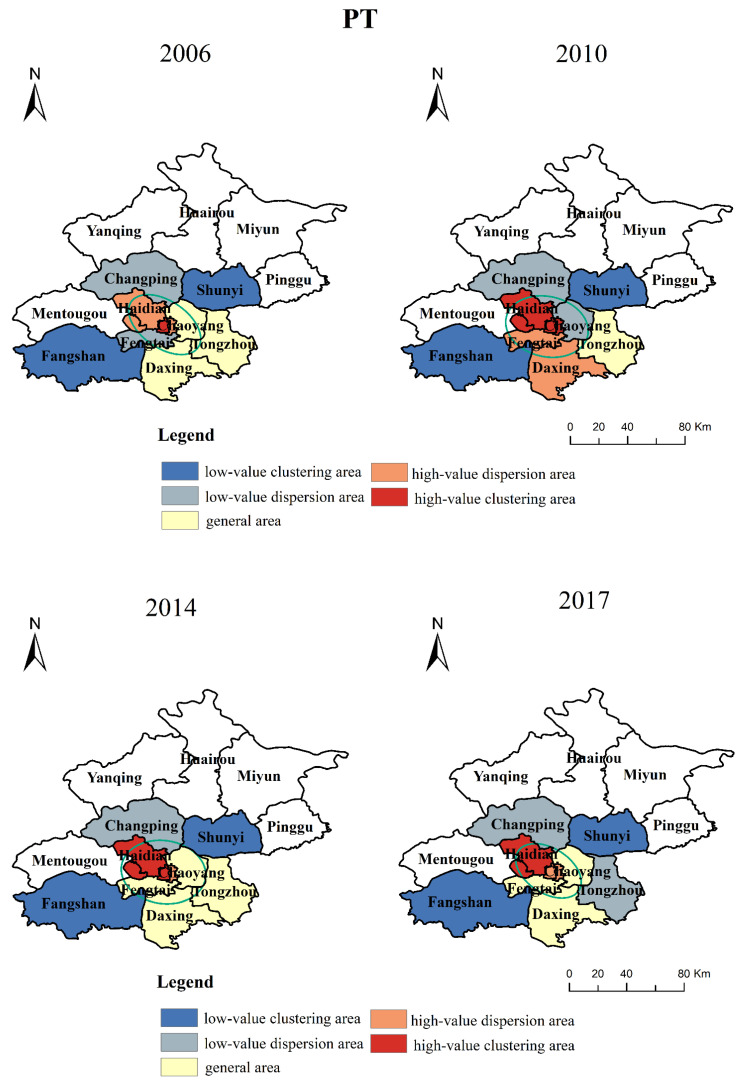

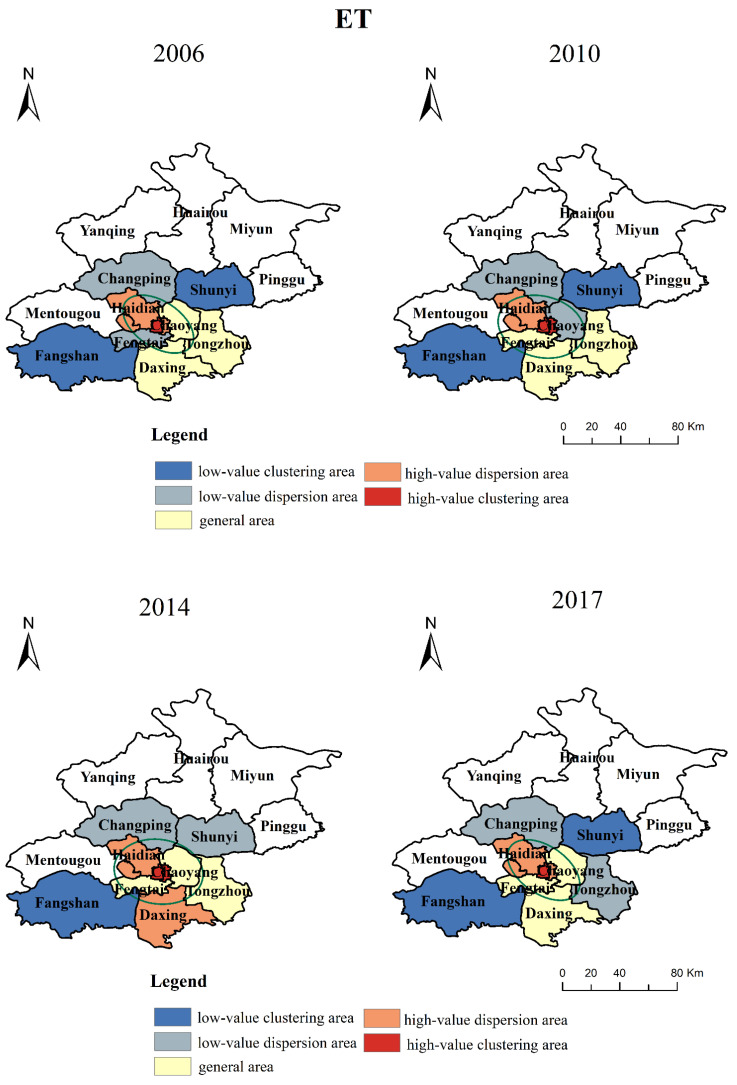

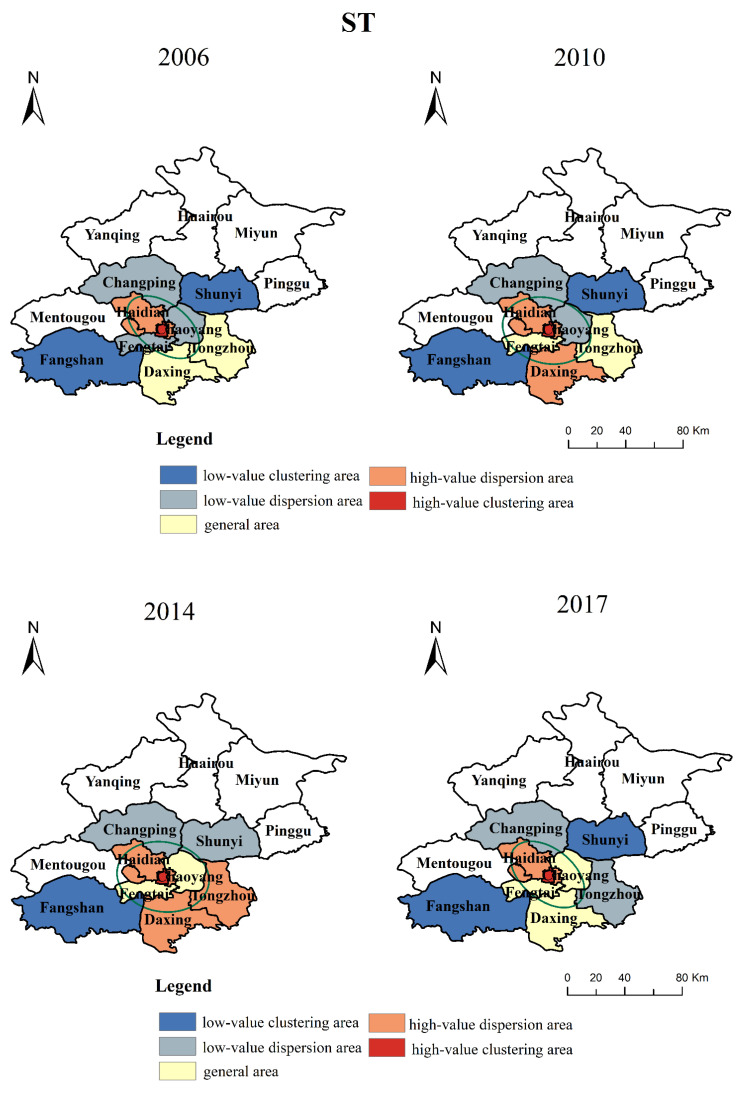
The spatiotemporal distribution of high–low value clustering points with different pairwise coupling coordination degrees.

According to Equations (11) and (12), we can get the spatiotemporal distribution of high–low value clustering points with different pairwise coupling coordination degrees, as shown in Figure 6.

Overall, from 2006 to 2017, the high-value dispersion and clustering areas of PT and ST are increased from four to six, and the high-value dispersion and clustering areas of ET are four and five, which means that the coupling coordination degree of rail transit and urbanization has a significant regional imbalance.

In various regions, Haidian, Dongcheng, Xicheng, and Shijingshan are high-value clustering and dispersion areas, which are the areas with a high degree of coupling coordination of RU and RT. Daxing, Chaoyang, Fengtai, and Tongzhou are general areas of coupling coordination degree, Shunyi, Changping, and Fangshan are low-value dispersion and clustering areas of coupling coordination degree. This regional imbalance results from the following: First, the urban rail construction in the central urban area is perfect, while the rail construction in the outer suburbs is relatively backward. By 2018, a total of 22 subway lines had been opened in Beijing, while there were only two subway lines to Fangshan. Second, the urbanization development level of the central urban area is significantly higher than that of the outer suburbs. For example, the GDP and per-capita disposable income of the central urban area is about 1.62 times and 1.12 times that of the outer suburbs.

#### 4.4.3. Analysis for Spatial Pattern Evolution of the Coupling Coordination Degree

**Figure 7:**
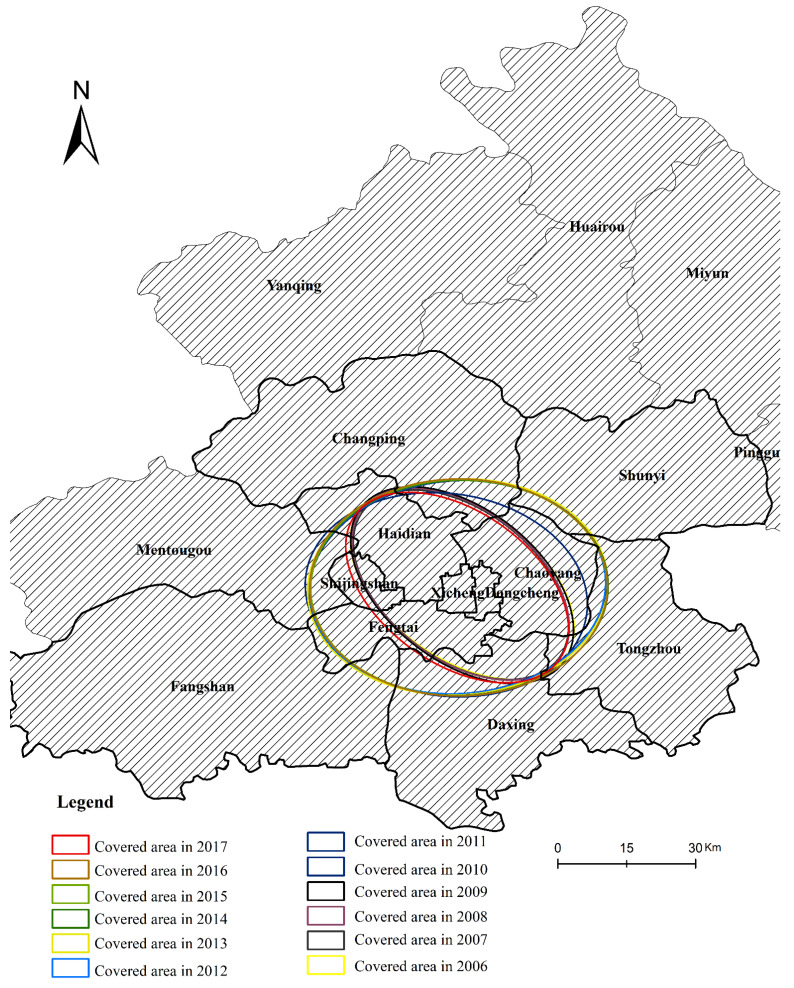
Evolution of the spatial distribution center and scope of overall coupling coordination degree in Beijing from 2006 to 2017.

As the standard ellipse spatial evolution distribution of the coupling coordination between RT and RU (which include overall coupling and pairwise coupling) shows a consistent trend of change, we take the ellipse spatial evolution distribution of the overall coupling coordination as an example for analysis and use ArcGIS for visualization, as shown in Figure 7. The standard deviation ellipse spatial evolution distribution of coupling coordination and spatial autocorrelation between RT and RU is shown in Figure 3, Figure 4, Figure 5 and Figure 6.

According to the standard deviation ellipse spatial evolution distribution of coordination degree between RT and RU, the coordination degree of RT and RU generally presents a northwest to southeast evolution pattern from 2006 to 2017, and the distribution area is mainly in the six districts (Dongcheng, Xicheng, Haidian, Chaoyang, Shijingshan, and Fengtai). It can be seen that the outer suburbs of Beijing will become the main body of improving the coordination between RT and RU.

From Figure 7, we can also find that from 2006 to 2017, the spatial distribution range and dispersion of coordination degree between RT and RU have been shrinking. The elliptic flatness gradually increases, indicating that the coordination degree between RT and RU has more and more obvious directional characteristics. This is because with the continuous improvement and construction of rail transit lines in 2010, the coordination degree of the six urban districts is significantly higher than that of the outer suburbs. Therefore, as the coordination degree of RT and RU evolves from northwest to southeast, the oblateness of the ellipse gradually increases.

From 2006 to 2017, the ellipse center shows a trend of developing from northwest to southeast, and the distribution area is mainly concentrated in the central urban area. Specifically, the ellipse coverage area is also shrinking, and the distribution center and coverage area of the coordinated degree of RT and RU are gradually spreading to the southeast. In terms of spatial distribution, Dongcheng, Xicheng, Haidian, Chaoyang, Shijingshan, and Fengtai have the highest coordination degree between RT and RU. This is because from 2006 to 2017, the coordination degree of RT and RU in this region is the highest, and the spatial pattern evolution characteristics also show a relatively unstable trend, which is manifested as northwest to southeast. Meanwhile, from 2006 to 2017, Changping, Shunyi, Fangshan, Tongzhou, and Daxing are the low-value distribution areas of the coordination degree between RT and RU, so they are always outside the coverage range of the standard deviation ellipse. This is because these regions are an exurb urban area, the urban rail opening is late, and the rail line is relatively imperfect, so the coordination degree of RT and RU in these regions is low, so the coverage of the standard deviation ellipse is relatively small.

## 5. Discussion

Compared with previous studies, our innovations mainly include the following three aspects. Firstly, based on the coupling coordination degree model and spatial autocorrelation model, we study and analyze the coordination relationship between rail transit and urbanization (population urbanization, economic urbanization, and spatial urbanization). Previous studies mainly focused on the relationship between urban spatial structure and rail transit, or the influence of population, land, and other factors on urbanization, but there were few studies on the relationship between rail transit and population, economy, and spatial urbanization. From the perspectives of population urbanization, economic urbanization, and spatial urbanization, we study the coordination relationship between them and rail transit respectively, and we analyze the causes of imbalance among different regions. Finally, based on the spatial autocorrelation model and standard deviation ellipse model, we study and analyze the spatial correlation and spatial evolution characteristic of coupling coordination degree among rail transit, population urbanization, economy urbanization, and spatial urbanization, thus further understanding the imbalance of rail transit and urbanization in different regions.

Secondly, based on the urbanization and rail transit data of 11 administrative regions of Beijing from 2006 to 2017, we measure the coupling coordination degree and spatial correlation between rail transit and urbanization in different regions. Most of the previous studies on urbanization have studied the causes of imbalanced urbanization development among different cities from the perspective of the city as a whole. Few studies have analyzed the factors affecting the imbalance of urbanization and the coordination relationship between rail transit and urbanization from the perspective of the urban inner region. The results show that the coupling coordination degree between urbanization and rail transit presents an imbalanced distribution in various regions, and the coupling coordination degree in the central city is significantly higher than that in the outer suburbs. By studying the coupling coordination between rail transit and urbanization in different regions, we find the relationship between rail transit and urbanization. For example, the coupling coordination degree of suburbs is low, so it provides theoretical guidance for the suburbanization of rail transit, the development of commuter rail, and the development of suburban railway.

Thirdly, we explore the internal causes of the imbalance of urban regional development from the coordination between rail transit and urbanization. From the perspective of time, different urbanization indicators and rail transit are in different trends. For example, from 2006 to 2017, the level of economic urbanization, spatial urbanization, and rail transit all showed a downward–upward–down trend, and the level of economic urbanization is the highest. From the perspective of space, the coupling coordination degree between rail transit and urbanization in different areas of the city is in an unbalanced state, but the spatial correlation of coupling coordination degree between each system has a similar trend. The coupling coordination degree of the central urban area is significantly higher than that of the outer suburbs, and the clustering points of high and low values are decreasing from the central urban area to the surrounding urban area. Therefore, it is necessary to strengthen the construction of suburban rail transit, develop the subcenter of the city, build a new suburbanization development model of urban space structure, and guide the orderly and compact expansion of urban space to achieve sustainable development of the city.

## 6. Conclusions

The coordination relationship between rail transit and urbanization development level is related to the sustainable development of the whole city, among which the imbalanced development of urban regions has gradually attracted attention. Taking Beijing as an example, we use the data of rail transit and urbanization from 2006 to 2017 to build a coupling coordination degree model. Finally, based on the spatial autocorrelation model, we analyze the spatial aggregation of coupling coordination degree. The specific conclusions are as follows:

Firstly, except for population urbanization, economic urbanization, spatial urbanization, and the rail transit system level have the same trend, and economic urbanization is at the highest level. On the whole, the coupling coordination degree between RU and RT is in a state of disorder, and the coupling coordination degree between various systems presents a consistent trend of change. The coupling coordination degree between EU and RT is at the highest level, while the coupling coordination degree between SU and RT is at the lowest level.

Secondly, the coupling coordination degree between RU and RT is imbalanced in each region, and the coupling coordination degree in the central urban area is significantly higher than that in the outer suburbs. On the whole, from 2006 to 2014, there is no significant change in the overall coupling coordination degree in the central urban area, but the coupling coordination degree in the outer suburbs (Changping, Shunyi, Tongzhou, Daxing, Fangshan) shows an obvious upward trend. In addition, the degree of coupling coordination in all regions significantly decreases in 2017. In addition, from the perspective of the spatial evolution pattern of the standard deviational ellipse, the distribution of the overall coupling coordination degree between 2006 and 2017 is consistent, mainly in the central urban area, Changping, and Shunyi.

Finally, on the whole, from 2006 to 2017, the coupling coordination degree between RU and RT has a similar trend in spatial correlation, and the Moran’s I values show a trend of decreasing first and then increasing. The high-value and low-value aggregation points of coupling coordination degree among the population, economy, and spatial urbanization and rail transit are similar, which shows a decreasing trend from the central urban to suburban areas.

In summary, it is found that the coordination relationship between urbanization and rail transit has an obvious regional imbalance. Relevant departments can formulate corresponding urban development planning strategies and urban transportation policies according to the urbanization development level of different regions and the coupling coordination degree and spatial aggregation degree between RU and RT to realize the healthy, green, and sustainable development of urban transportation.

Our research has some limitations at present. Firstly, the selection of evaluation indicators for RT and RU mainly relies on the previous literature indicator system and the existing statistical data of Beijing. Therefore, our selection of the indicators of RU and RT is not comprehensive; we need more open source data to support the selection of other indicators: for example, rail transit lines in different areas of the range, a single line of rail passenger flow data and residents’ commuting times, the total number of passengers, the train service quantity and train departure frequency, and so on. Secondly, limited by the availability of data, the multi-source data used in this paper are not perfect, such as the number of passengers in each rail line. Moreover, some social factors, such as local policies and data openness, make it difficult to obtain and quantify some data. As the city is a complex system, the data of the urban development level are formed by the interaction of complex data of multiple dimensions, which has the characteristics of multiple heterogeneity. Therefore, this paper only studies the coordination relationship between RT and RU from 2006 to 2017, and it does not expand the research period, which is not conducive to a more accurate and comprehensive grasp of the interaction between urbanization and urban rail and its change law. In the future, we can try to make innovations in the research factors, use multi-source data to supplement the research on the relationship between urbanization development level and rail transit, and analyze the factors affecting urbanization development and urban transportation from multiple dimensions and dividing scales. In addition, the influence of different public transport systems on urbanization is also worth further exploration.

## Figures and Tables

**Table 1:** Representative studies on influencing factors of rail transit and urbanization.

Research Topic	Study Authors	Research Content	Research Methods	Research Process
The interrelation between rail transit and urbanization	Hou [23]	Coordination relationship between rail transit and land use	Data envelopment analysis (DEA) and clustering method	It is found that the relationship between rail transit capacity and land use in high population density rail transit stations is imbalanced
	Liu [24]	Coordination between rail transit and land use system	Utility function, coupling coordination model	The comprehensive development level of rail transit system and land use system is calculated, and the coordination of Shanghai rail transit and land use system is quantitatively analyzed
	Cai [25]	Combined with the foreign urban rail transit network system, propose urban rail transit models and corresponding technical recommendations	Urban rail transit model	This paper analyzes the urban spatial structure and land distribution characteristics, puts forward the urban rail transit model, and puts forward the corresponding technical suggestions based on the foreign urban rail transit network system and the corresponding connection mode
	Cai [26], Feng [27]	The influence of rail transit on urban spatial structure, land use, and spatial quality	Space coupling model	This paper expounds the principle of spatial coupling from the perspective of spatial overlap between rail transit stations and urban center system, and points out that rail transit will have a significant or fundamental impact on urban spatial structure, land use, and spatial quality
Related factors affecting the urbanization	Xia [35]	Coupling coordination relationship between urban function mixing and urbanization level	Information entropy and coupling coordination model	Based on multi-source data and information entropy and coupling coordination degree model, this paper analyzes the coordination relationship between the urban functional mixing state and urbanization development level and its influencing factors from the perspective of urban spatial structure development
	Cao [36]	Coordination of population, land, and economic urbanization	System theory	The coordination of population, land, and economic urbanization is studied from the perspective of system theory
	Zhang [37]	Coordination of urban quality and scale	Quadrant method	This paper makes a quantitative study on the coordination between urbanization quality and scale in Jiangsu Province by using the quadrant method
	Li [38]	Coordination between land, population, and industrial urbanization	Coupling coordination model	The coupling model is used to study the coordination of urbanization in Chongqing from three dimensions of land, population, and industry
	Li [39]	Coordination of urbanization in urban, industrial cities, regional urbanization, and resource environment	Theil coefficient	The Theil coefficient is used to measure the urbanization coordination of 105 prefecture-level cities in the Yangtze River Economic Belt from four aspects: urban and rural, industrial city, regional urbanization, and resource environment
	Tian [40]	Temporal and space coupling and interaction between ecosystems and urbanization	Coupling coordination degree model and spatial autocorrelation statistical model	Coupling coordination degree model and spatial autocorrelation statistical model are used to evaluate the spatiotemporal coupling and interaction between ecosystem and urbanization, and to understand the coupling relationship between urbanization and ecological protection
	Li [41], Wang [42]	Coordination relationship between urbanization level and ecological environment	Coupling coordination degree model	The relationship between urbanization level and ecological environment is analyzed, and it is found that the coupling coordination degree between urbanization level and ecological environment presents a U-shaped curve over time [40]. The study finds that the coupling coordination degree between population urbanization and ecological environment in Beijing–Tianjin–Hebei presents an S-shaped curve over time [41]
	Zhao [43]	The relationship between compactness and urbanization	Compactness measurement, GWR model, and spatial autocorrelation	A new compactness measurement method is proposed, and the relationship between compactness and urbanization is discussed by using the GWR model and spatial autocorrelation analysis
	Ma [44]	The development coordination degree of different cities is analyzed from three aspects of economy, society, and ecological space	Coupling coordination degree model	This paper analyzes the development coordination degree of cities in the middle reaches of the Yangtze River from three aspects of economy, society, and ecological space, and it analyzes the differences among different regions

**Table 2:** Evaluation indicator systems.

Criteria Layer	Indicator Layer	Unit	Weight
Population urbanization	Number of employees in urban units	number	0.039
	Permanent population density	number/km^2^	0.043
	Number of civilian cars per 10,000 people	number	0.044
	Number of private cars per 10,000 people	number	0.046
Economic urbanization	Average salary of urban employees	yuan	0.040
	Urban disposable income per capita	yuan	0.052
	Local fiscal expenditure	10 thousand yuan	0.017
	Local fiscal revenue	10 thousand yuan	0.043
	GDP	10 thousand yuan	0.091
	Primary industry output value	10 thousand yuan	0.051
	Secondary industry output value	10 thousand yuan	0.032
	Tertiary industry output value	10 thousand yuan	0.058
	Total wholesale and retail sales	billion	0.053
	Fixed asset investment	billion	0.040
	Average house price	yuan	0.026
Spatial urbanization	Number of ordinary secondary schools	number	0.017
	Number of hospitals	number	0.049
	Floor space completed	10 thousand square meters	0.037
	Commercial housing sales area	10 thousand square meters	0.038
Rail transit	Number of stations	number	0.074
	Metro lines serving the area	number	0.058
	Whether the main suburban line that serves it is directly connected to the city center, whether it enters Line 10	/	0.051

Note: Private cars refer to the buyer as private, individual. While civilian cars belong to the public, the purchasers are entities, companies, troops, and enterprises. The premise of classification is different.

**Table 3:** Classification standard of coupling degree.

Range	Coupling Level	System Relationship
C=0	No coupling	Unrelated
0<C<0.4	Low-level coupling	Extremely unstable
0.4≤C<0.6	Medium level	Unstable
0.6≤C<0.8	Relatively high level	Basically stable
0.8≤C<1	The highest level	Very stable

**Table 4:** Evaluation criteria of coupling coordination degree.

**Degree of Coordination**	**0–0.009**	**0.10–0.19**	**0.20–0.29**	**0.30–0.39**	**0.40–0.49**
Coordination situation	Extreme disorder	Serious disorder	Medium disorder	Minor disorder	Barely disorder
**Degree of Coordination**	**0.50–0.59**	**0.60–0.69**	**0.70–0.79**	**0.80–0.89**	**0.90–1.00**
Coordination situation	Barely coordination	Primary coordination	Medium coordination	Good coordination	Extreme coordination

**Table 5:** Coupling degree between urbanization and rail transit.

Year	C	$C^{PT}$	$C^{ET}$	$C^{ST}$
2006	0.660	0.758	0.763	0.688
2007	0.646	0.721	0.768	0.683
2008	0.659	0.741	0.763	0.714
2009	0.652	0.735	0.775	0.700
2010	0.722	0.830	0.831	0.785
2011	0.769	0.885	0.858	0.843
2012	0.774	0.881	0.858	0.835
2013	0.783	0.882	0.859	0.832
2014	0.758	0.871	0.844	0.813
2015	0.758	0.871	0.839	0.816
2016	0.768	0.874	0.842	0.819
2017	0.620	0.660	0.767	0.736

**Table 6:** The global autocorrelation of coupling coordination degree.

Year	D		$D^{PT}$		$D^{ET}$		$D^{ST}$	
	*Moran’s I*	*Z*(*I*)	*Moran’s I*	*Z*(*I*)	*Moran’s I*	*Z*(*I*)	*Moran’s I*	*Z*(*I*)
2006	0.145	1.704	0.232	2.319	0.248	2.425	0.082	1.270
2007	0.166	1.850	0.279	2.638	0.248	2.423	0.064	1.146
2008	0.138	1.660	0.229	2.294	0.200	2.091	0.047	1.025
2009	0.096	1.363	0.167	1.862	0.194	2.049	0.021	0.843
2010	0.034	0.937	0.194	2.053	0.102	1.417	−0.088	0.085
2011	0.020	0.557	0.178	1.925	0.057	1.089	−0.12	−0.142
2012	−0.025	0.523	0.18	1.938	0.064	1.137	−0.136	−0.250
2013	−0.032	0.476	0.185	1.973	0.048	1.026	−0.141	−0.287
2014	−0.029	0.494	0.217	2.201	0.055	1.078	−0.133	−0.232
2015	0.009	0.759	0.225	2.255	0.103	1.409	−0.116	−0.110
2016	−0.002	0.684	0.230	2.293	0.089	1.316	−0.133	−0.233
2017	0.164	1.838	0.303	2.796	0.101	1.404	−0.027	0.506

## Data Availability

The data used to support the findings of this study are available from the corresponding author upon request.
